# Insights into the mechanism of a G-quadruplex-unwinding DEAH-box helicase

**DOI:** 10.1093/nar/gkv051

**Published:** 2015-02-04

**Authors:** Michael C. Chen, Pierre Murat, Keren Abecassis, Adrian R. Ferré-D'Amaré, Shankar Balasubramanian

**Affiliations:** 1 Department of Chemistry, University of Cambridge, Lensfield Road, Cambridge, CB2 1EW, UK; 2 National Heart, Lung and Blood Institute, 50 South Drive, MSC-8012, Bethesda, MD 20892–8012, USA; 3 Cancer Research UK Cambridge Institute, Li Ka Shing Centre, Cambridge, UK

## Abstract

The unwinding of nucleic acid secondary structures within cells is crucial to maintain genomic integrity and prevent abortive transcription and translation initiation. DHX36, also known as RHAU or G4R1, is a DEAH-box ATP-dependent helicase highly specific for DNA and RNA G-quadruplexes (G4s). A fundamental mechanistic understanding of the interaction between helicases and their G4 substrates is important to elucidate G4 biology and pave the way toward G4-targeted therapies. Here we analyze how the thermodynamic stability of G4 substrates affects binding and unwinding by DHX36. We modulated the stability of the G4 substrates by varying the sequence and the number of G-tetrads and by using small, G4-stabilizing molecules. We found an inverse correlation between the thermodynamic stability of the G4 substrates and rates of unwinding by DHX36. In stark contrast, the ATPase activity of the helicase was largely independent of substrate stability pointing toward a decoupling mechanism akin to what has been observed for many double-stranded DEAD-box RNA helicases. Our study provides the first evidence that DHX36 uses a local, non-processive mechanism to unwind G4 substrates, reminiscent of that of eukaryotic initiation factor 4A (eIF4A) on double-stranded substrates.

## INTRODUCTION

G-quadruplexes (G4s) are a class of four-stranded secondary structures that can form within guanine-rich nucleic acids (1). Such structures were recently detected by immunofluorescnce in the nucleus (DNA (2)) and cytoplasm (RNA (3)) of human cells and tissue (3). DHX36 is a member of the DEAH-box helicase family (4) and exhibits adenosine triphosphate (ATP)-dependent, G4-specific unwinding activity *in vitro* (5,6). DHX36 was identified as the major source of RNA G4-unwinding activity in HeLa (human) cell lysate (7). Moreover, the expression of some G4-containing genes was shown to be regulated by DHX36 in human cells. YY1 (8) and PITX1 (9), two genes involved in cancer biogenesis, are notable examples of DHX36 regulation and highlight the role of DHX36 as a potential target for therapeutic intervention. *In vitro* characterization of DHX36 revealed that the helicase binds G4 substrates with remarkably low, picomolar dissociation constants and only unwinds DNA and RNA G4s (6). Other members of the DEAH-box helicase family have been shown to target other non-double-stranded (ds) nucleic acid structures such as triplexes and ribonucleoprotein complexes (10). Analysis of how DHX36 recognizes and unwinds G4 is thus key to our understanding of how helicases process non-canonical nucleic acid structures (11).

While the mechanism by which DHX36 and its close homologs, such as DHX9 (12), recognize and unwind G4 structures remains uncharacterized, this process is better understood for the closely related DEAD-box family of helicases, which unwind double-stranded RNA substrates (13). For example, two mechanistic properties of eIF4A and DED1, both typical DEAD-box helicases (14), are their high sensitivity to substrate thermodynamic stability and decoupled ATP consumption (14–17). More specifically, the unwinding rate of DEAD-box enzymes inversely correlates with the thermodynamic stability of ds substrates as evident by the decreased activity of eIF4A for dsRNA with a higher GC content (15). Furthermore, even though helicase-catalyzed strand separation is dependent on ATP, not every ATPase cycle results in strand separation. This decoupling reflects the non-processive (18) and local (19) nature of all DEAD-box helicases, which dissociate from their nucleic acid substrates after each ATP hydrolysis event.

Decoupled ATP hydrolysis has been previously observed for G4 helicases more distant to DHX36. The processive, translocating RecQ helicase, BLM—the dysfunction of which is implicated in Bloom syndrome—was shown to unwind G4 *in vitro* (20). The BLM helicase displayed an ATP hydrolysis rate that was independent of substrate stability in the presence of a small molecule ligand, N-methyl mesoporphyrin IX (NMM) (21). The decoupling was proposed to be the result of a stalled BLM helicase, which consumed ATP but was unable to unwind the G4 substrate (21).

To gain insight into the mechanism by which DEAH-box helicases unwind G4 structures, we studied the effect of thermal stability of G4-templates on the binding and unwinding activities of DHX36. By adjusting substrate stability either by varying the number of G-tracts or by binding of G4-stabilizing ligands, we demonstrate DHX36 unwinding is highly sensitive to substrate stability. Furthermore, we find that ATP consumption by DHX36 is largely independent of G4 stability. Together, we propose that DHX36 unwinds G4 nucleic acids using a mechanism similar to that employed by homologous DEAD-box helicases that act on double-stranded nucleic acids.

## MATERIALS AND METHODS

### Circular dichroism (CD) spectroscopy

CD spectroscopy was used to evaluate G4 topology and thermal stability (22). CD spectra were obtained on samples in 1 mm path length cuvettes using a Chirascan-plus CD spectrometer. Spectra were obtained in the previously reported K-Res buffer (5) (50 mM Tris acetate pH 7.8, 100 mM KCl, 10 mM NaCl, 70 mM glycine, 10% glycerol, 3.3 mM MgCl_2_, 0.12% bovine α-lactalbumin (Sigma), 10 ng·μl^−1^ poly dI:dC (Sigma)) without bovine α-lactalbumin or poly(dI:dC). G-quadruplex forming oligonucleotides (5G-8G, Z33) were annealed in 10 mM Tris-HCl (pH 8.0) and 100 mM KCl at a stock concentration of 100 μM, then diluted at a 10μM concentration in K-Res buffer for further analysis. Scans were performed over the range of 210–350 nm at variable temperatures for melting experiments. Each trace is the result of a step size of 1 nm, a time per point of 1 s and a bandwidth of 1 nm. Melting temperatures were determined in triplicate using the Applied Photophysics Global3 Analysis Program software.

### Crystallization and structural determination of r(UGGGGU)_4_ tetramolecular G4

**Figure 1.**
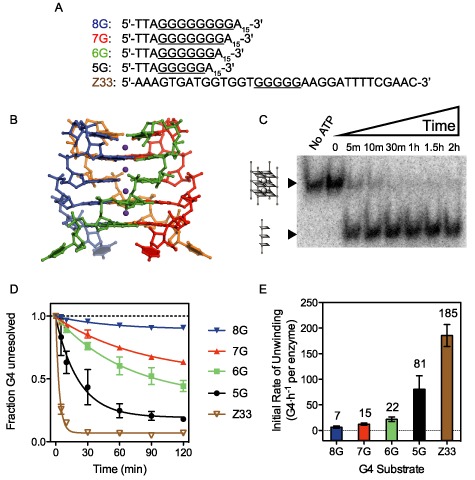
(**A**) Sequences of the DNA intermolecular G4s used as substrates for DHX36. All strands were annealed in solutions containing potassium to form G4s prior to use. (**B**) A tetramolecular RNA G-quadruplex (G4) whose structure was elucidated by X-ray crystallography in this study. The RNA G4 (5′-UGGGGU-3′) adopts a parallel topology typical of the intermolecular G4s used as substrates for DHX36 (PDBID 4XK0). (**C**) Helicase activity assay time course. Recombinant, full-length DHX36 (110 pM) was incubated with radiolabeled Z33 G4 (4 nM) in K-Res buffer and the reaction was stopped at the indicated times with proteinase K. The ratio of single-stranded and folded G4 DNA was analyzed by native PAGE. (**D**) Resolution of Z33 and 5G-8G intermolecular G4s by DHX36; experiments performed identically to that shown in (C). (**E**) Initial rate of unwinding of G4 in the first 10 min of DHX36-mediated G4 resolution, as shown in (D). All experiments were performed in triplicate; error bars represent standard deviations.

Crystals of the 5′—r(UGGGGU)—3′ (GE-Dharmacon) G-quadruplex were obtained by vapor-diffusion at 21°C. Hanging drops were prepared by mixing a 2 mM (on a strand basis) solution of the RNA, also containing 10 mM HEPES KOH (pH 7.5) and 100 mM KCl, in a 1:1 ratio with the reservoir solution (40 mM potassium cacodylate (pH 6.0), 35% 2-methyl-2,4-pentanediol, 5 mM spermine hydrochloride, 80 mM potassium chloride, 0.5 mM DHX36 fragment and 20 mM barium chloride). Crystals grew over three months to maximum dimensions of 0.5 × 0.5 × 0.1 mm^3^. Crystals were directly mounted on nylon loops and vitrified by plunging into liquid nitrogen. Single-wavelength anomalous dispersion (SAD) data were collected at 100 K with 0.8266 Å X-radiation at beamline 5.0.2 of the Advanced Light Source (ALS). Diffraction data were indexed, integrated, and scaled with HKL2000 (23). Data collection statistics are summarized in Supplementary Table S1. After data preparation with SHELXC, the best substructure was found in SHELXD (24) with default parameters using a data cutoff of 1.3 Å and searching for 12 marker atoms. Six sites were used as input for phenix.autosol (25) with a resolution cutoff of 1.08 Å, yielding a mean overall figure of merit of 0.687. The final SAD heavy atom substructure consisted of 4 barium ions, 8 potassium ions and 2 phosphorus atoms. The resulting electron density map without density modification allowed initial tracing of RNA using COOT (26). Refinement was performed using phenix.refine (25). The crystallographic asymmetric unit consists of two RNA strands, 12 K^+^ ions (at an occupancy of 0.25), 3 Ba^2+^ ions and 62 water molecules. Figures 1B and S2 were prepared with PyMOL (http://www.pymol.org).

### DHX36 expression and purification

Recombinant DHX36 was expressed and purified (Supplementary Figure S3) according to procedures similar to those already reported (7). This DHX36 construct contains all the residues found in isoform 1 (GenBank NP_065916.2) and incorporates a 6×His-tag fused directly to the N-terminal end.

### DHX36 binding assays

Oligonucleotides (Invitrogen) at a concentration of 100 μM were annealed to form G4s by heating at 99°C for 20 min and slowly cooling to 4°C over several hours in 10 mM Tris-HCl (8.0) and 100 mM KCl. Oligonucleotides analyzed by polyacrylamide gel electrophoresis (PAGE) were radiolabeled following standard protocols using T4 polynucleotide kinase and [γ-^32^P] ATP. Oligos were then purified using ProbeQuant G-50 microspin columns (GE). DHX36 was incubated for 30 min at 37°C with 10 pM radiolabeled, annealed Z33 G4 in K-Res buffer supplemented with 10 mM EDTA in a volume of 50 μl. Following incubation, the reaction was directly loaded onto a 6% polyacrylamide gel (37.5:1) (National Diagnostics) and run at 30 mA for 1.5 h. When binding inhibition was studied through the use of a ligand, ligand was added at indicated concentrations after the initial 30 min incubation. Experiments with ligand titration contained 4 nM DHX36 and 4 nM Z33 G4. The mixture was then incubated for an additional 10 min at 37°C before being analyzed by PAGE. Gels were either directly imaged or dried using a BioRad Model 583 Gel Drier and exposed to a storage phosphor screen followed by visualization with a Typhoon Trio scanner (GE). Bands were quantified using ImageQuant TL software. All ligand binding data were fit with three-phase exponential decay functions.

### DHX36 unwinding assays

DHX36 (110 pM) was incubated for the indicated amounts of time at 37°C with 4 nM annealed G4 (0.08% radiolabeled) in the K-Res buffer (50 mM Tris acetate pH 7.8, 100 mM KCl, 10 mM NaCl, 70 mM glycine, 10% glycerol, 3.3 mM MgCl_2_, 0.12% bovine α-lactalbumin (Sigma), 10 ng·μl^−1^ poly(dI:dC) (Sigma)) supplemented with 1 mM ATP in a volume of 20 μl. Reactions were stopped with proteinase K (20 mg·ml^−1^, 5 μl, Applied Biosystems) followed by incubation at 37°C for 10 min. Reactions were directly loaded onto a 20% poly acrylamide gel (19:1) and run at 350 V for 2.5 h. Gels were handled as described above. Only bands corresponding to the unimolecular (unfolded) and tetramolecular (folded) species were considered for quantification. Initial rates of unwinding were determined by performing a linear regression of the first 10 min of fraction G4 unwound curves. All unwinding assay data plotted as a function of time were fit with one-phase exponential functions. Unpaired Student's *t*-test was used to analyze initial unwinding rate significance.

### Modulation of DHX36 activity with small molecule ligands

Ligands **1–4** (pyridostatin (PDS), Phen-DC3, 12459, **4**) were dissolved in 50% DMSO stocks at 10 mM and directly diluted for use in enzymatic assays. The synthesis and characterization of all compounds used in this paper were previously reported (see text for references). Unwinding and binding inhibition assays were fit with exponential decay functions.

### DHX36 ATPase consumption assays

DHX36 was incubated in K-Res buffer with 0.01 mM ATP spiked with 50 nCi [γ-^32^P]-ATP in the presence of either poly(U) (100 ng·μl^−1^, Sigma) or 100 nM Z33 G4 and 5 μM ligand. At indicated times, reactions were stopped by adding EDTA (0.5 M, 1 μl, pH 8.0) to a 5 μl aliquot of reaction. ATP hydrolysis was quantified by CEL PEI/UV254 TLC plates (Polygram) in 0.8 M acetic acid/0.8 M LiCl aqueous running solution after washing in methanol. TLC plates were air dried. Imaging was as for PAGE. Hydrolysis data were fit with one-phase exponential decay. One-way ANOVA was used to assess the significance of ATP hydrolysis data.

## RESULTS

### Increased G4 thermal stability reduces DHX36 G4-unwinding activity

To elucidate the effect of thermodynamic stability of G4-substrates on DHX36 helicase activity, we first studied different tetramolecular quadruplex substrates of the form [TTAG_n_A_15_]_4_, that differ only in the number (n = 5 through 8, denoted ‘5G’ through ‘8G’) of tetrad-forming guanines, as well as the Z33 G-quadruplex (Figure 1A), derived from a mutational hotspot in the tRNA supF gene (27). Tetramolecular G4 substrates were chosen for their kinetic stability and straightforward characterization through native PAGE (28). Tetramolecular G4s are commonly used for the study of protein or small molecule recognition of G4s (38,47–48). All tetramolecular G4 substrates were shown to adopt a parallel G4 topology by circular dichroism (CD) spectroscopy (Supplementary Figure S1). The core of the G4s is composed of stacked guanine tetrads as exemplified by the crystal structure of r[UGGGGU]_4_ presented in Figure 1B. The X-ray structure of r[UGGGGU]_4_, now determined at 1.08 Å resolution using crystals grown in the presence of the physiological cation, K^+^ (Figure 1B and Supplementary Figure S2 and Supplementary Table S1), confirms that it adopts a parallel, all *anti-*glycosidic conformation. (A structure of the same oligonucleotide forming a quadruplex bound to Sr^2+^ has been reported previously (29)). A 3′-tail was appended to each substrate to ensure productive loading of the helicase (5). DHX36 displays picomolar dissociation constants for these substrates and comparable affinity for all five tetramolecular G4 substrates used in this study (Supplementary Figures S3 and S4).

Since the formation of G4 structures is enthalpically driven, with a per-tetrad ΔΔ*H* of -20 to -25 kcal·mol^−1^ (30), increasing the number of guanine tetrads thus increases the thermodynamic stability of the formed G4. A PAGE-based helicase-unwinding assay (Figure 1C and Supplementary Figure S5) demonstrates that the fraction of unwound G4s (Figure 1D), and the initial rate of unwinding (Figure 1E) are both dependent on the number of guanine tetrads present on the substrate. Indeed, after 120 min under our experimental conditions, G-quadruplexes with 5 G-tetrads (5G and Z33) were completely unwound while >75% of the G-quadruplexes containing 8 tetrads (8G) were intact, a significant difference (*p* < 0.005, two-tailed). Initial rates of unwinding also follow the same trend, indicating that DHX36 G4-unwinding activity varies as a function of the stability of the G4 substrate.

Although not statistically significant (*p* > 0.05, two-tailed), differences in the initial rates of unwinding and the final unwound fractions of Z33 and 5G (Figure 1C and D) may suggest that the flanking sequences of the G4 could affect DHX36 activity. For example, flanking sequences may affect DHX36 loading on the G4-substrate in a sequence-specific manner. To separate this potentially confounding effect from the thermodynamic stability of G4 substrates we next modulated the stability of Z33 using external factors in the form of G4-stabilizing ligands.

### G4-stabilizing ligands reduce DHX36 G4-unwinding activity as a function of G4 thermal stability

**Figure 2.**
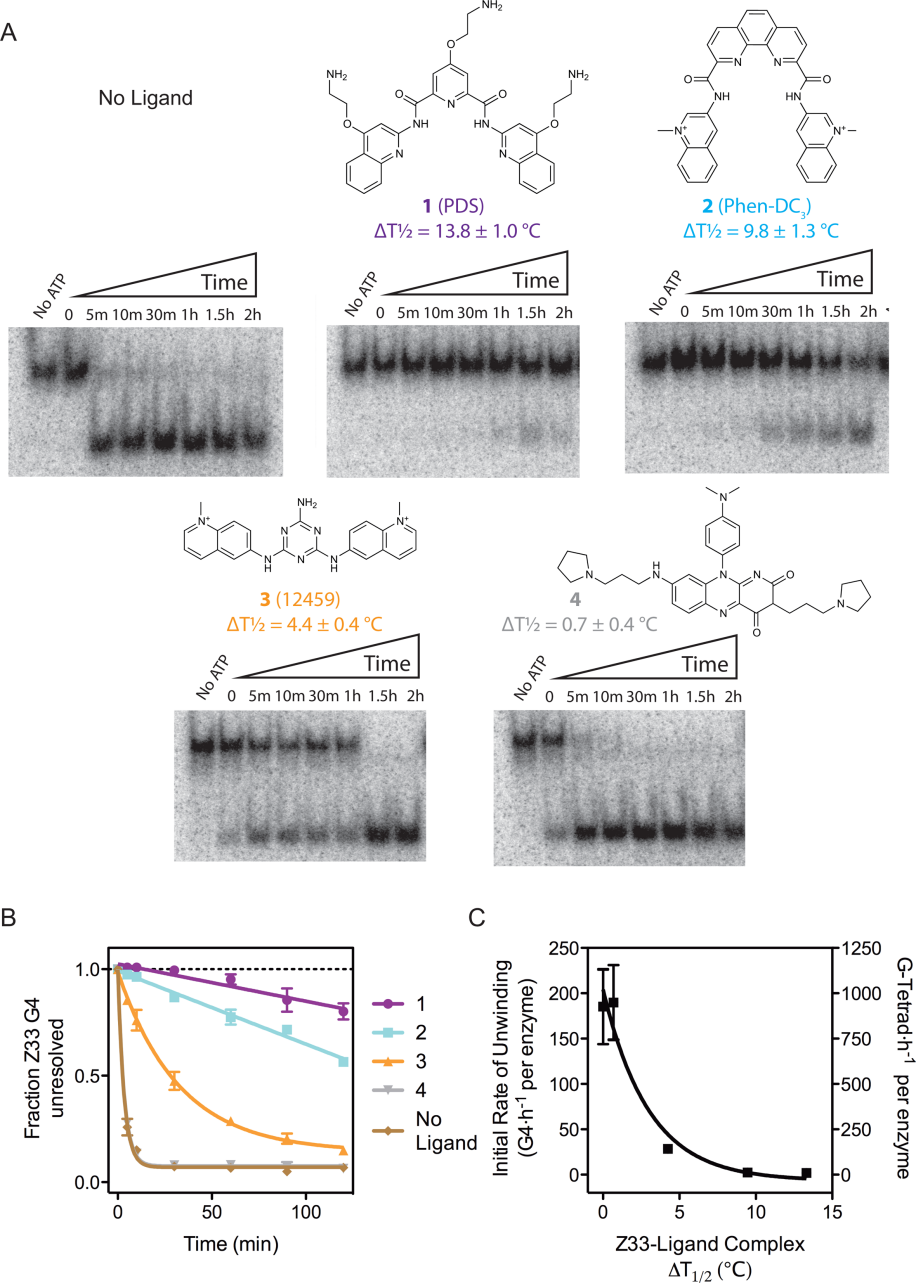
(**A**) Molecular structures of previously reported G4-stabilizing ligands used in this study. Differential transition temperature of the Z33 G4 with and without the presence of ligand is indicated below each respective structure. Polyacrylamide gels show the quantity of unwound G4 (higher electrophoretic mobility band) as a result of the addition of the corresponding G4-stabilizing ligand at 5 μM (above each gel). (**B**) Influence of the G4-stabilizing ligands on the unwinding activity of DHX36. DHX36 (110 pM) was incubated with radiolabeled Z33 G4 (4 nM) in K-Res buffer supplemented with ATP and stopped with proteinase K at indicated times. (**C**) Initial rate of G4 unwinding plotted as a function of Z33-ligand complex Δ*T*_1/2_. DHX36 is not permanently inhibited by the Z33-ligand interaction, but rather is retarded as the Z33-ligand Δ*T*_1/2_ increases. All experiments were performed in triplicate; error bars represent standard deviations.

From a library of small molecules, we chose four ligands for their varying ability to stabilize G4 substrates (Figure 2A). One chosen ligand, pyridostatin (PDS; **1**), was previously shown to inhibit binding of the shelterin component, POT1, to human telomeric DNA (31) as well as to induce DNA damage at G4 motifs in human cancer cells (see Figure 2A for structure) (32). The other ligands include Phen-DC3 (**2**) (33,34) and 12459 (**3**) (35), both of which were shown to bind G4 and inhibit telomerase activity, and **4**, which was shown to modulate the expression of a G4-containing proto-oncogene, *c-Kit* (36). We quantified their effect on Z33 G4 stability in thermal denaturation experiments monitored by CD spectroscopy. At a saturating, 5-fold molar excess of ligand over G4, the thermal stability of the ligand-G4 complex increased in the order of **1** (PDS) > **2** (Phen-DC3) > **3** (12459) > **4** > no ligand (Supplementary Figure S6, transition temperatures reported in Figure 2A). Through CD spectroscopy, we confirmed that binding of **1–4** does not substantially alter the topology of or unwind the G4 substrate (Supplementary Figure S1). Next, we performed DHX36 (110 pM) unwinding assays in the presence of ligand (5 μM) and Z33-G4 (4 nM), conditions under which each ligand is saturating (Figure 2A). We found that the apparent initial rates of G4 unwinding decreased as a function of thermal stability imparted by ligand binding (Figure 2B and C). Notably, in the presence of **1** G4 unwinding was reduced by 100-fold (Figure 2C). In comparison, **2** and **3** reduced G4 unwinding by 80-fold and 8-fold, respectively. Ligand **4** did not reduce G4 unwinding. Our results demonstrate that modulating G4 stability either by increasing G-tetrad quantity or by adding G4-stabilizing ligands results in a similar dose-dependent reduction in G4-unwinding.

To confirm that the initial rate of unwinding varies primarily as a function of thermal stability, we measured DHX36 binding to G4 in the presence of ligand, independently from helicase activity (Supplementary Figures S7 and S9). If ligand-induced changes in the association constant of DHX36 for its substrate were responsible for changes in the unwinding rates, then levels of binding inhibition should be proportional to the levels of unwinding inhibition. Otherwise, we reason that inhibition is due to the ligand-induced thermal stabilization of the G4 substrate. To quantify binding inhibition by ligand, DHX36 was first bound to Z33 G4, and the resulting complex was subsequently exposed to increasing concentrations of ligands (Supplementary Figure S7). While ligands **2** to **4** inhibit DHX36 substrate binding from 74% to 6% of control binding (when no ligand is present), the corresponding DHX36 G4 unwinding activity is non-proportionally inhibited from 22% to 1% of control activity, respectively. Additionally, the presence of **1** and **2** resulted in almost identical levels of DHX36 substrate binding (70% and 74% at a 5 μM concentration), whereas the initial rates in the helicase assay differ by more than two-fold (Supplementary Figures S8 and S9). Since the amount of binding inhibition observed in the presence of **1** and **2** is similar, but the amount of activity differs, we conclude that the decrease in unwinding activity is primarily attributed to differential substrate stability. The observation that the reduction in G4 unwinding activity in the presence of **1–4** does not correspond with binding inhibition supports the conclusion that unwinding rates mainly vary as a function of substrate thermal stability.

**Figure 3.**
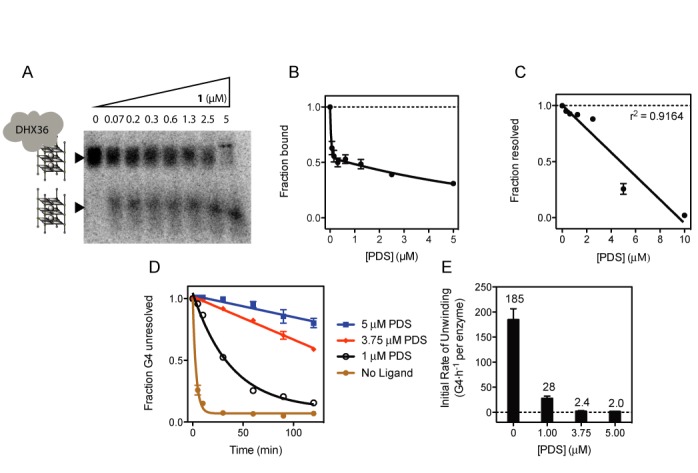
(**A**) Inhibition of DHX36 binding by **1** (PDS, Figure 2A). DHX36 (4 nM) was bound to radiolabeled Z33 G4 (4 nM) for 30 min in K-Res buffer without ATP. **1** was then titrated at the specified concentrations and DHX36 binding was quantified (**B**) by electrophoretic mobility shift analysis (EMSA). (**C**) Inhibition of DHX36 G4 resolution by **1**. DHX36 (110 pM) was incubated with radiolabeled Z33 G4 (4 nM) at specified concentrations of **1** in K-Res buffer and ATP for 1 h. Inhibition of DHX36 G4 resolution was quantified by native PAGE and fit with a linear model. (**D**) Retardation of DHX36 G4 resolution by PDS. DHX36 (750 pM) was incubated with Z33 G4 (4 nM) with and without PDS at indicated concentrations and stopped at the specified times with proteinase K. (**E**) Apparent initial rate of unwinding (number of G4 unwound per hour per enzyme) of Z33-PDS complex in the first 10 min of DHX36-mediated G4 resolution, as shown in (D). Numbers above bars represent the amount of G4 unwound per hour by each DHX36 helicase. All experiments represent triplicated samples; error bars represent ± standard deviation.

**Figure 4.**
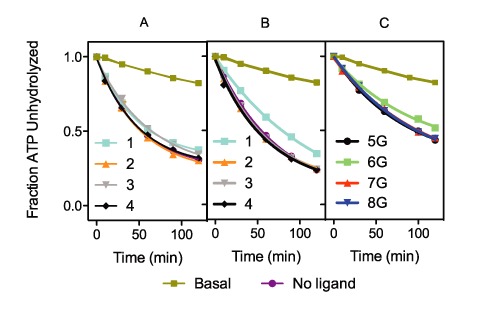
DHX36 (4 nM) ATP consumption in the presence of the ligands **1–4** and (**A**) poly (U) (100 ng·μl^−1^) or (**B**) Z33 G4 (100 nM) substrates. (**C**) DHX36 ATP consumption was measured with and without dTTAG_n_A_15_. DHX36 was incubated with the nucleic acid substrate, ligand (5 μM), and ATP (0.01 mM) spiked with [γ-32P]-ATP in K-Res buffer for 1 h. The reactions were stopped with proteinase K and analyzed by PEI-TLC (Supplementary Figures S11 and S12). All experiments performed in triplicate; error bars represent standard deviations.

We further isolated the effect on the rate of DHX36 G4 unwinding to differential ligand stabilization (Figure 2B) by titrating **1** in both binding and unwinding assays (Supplementary Figure S10). In the first set of experiments we determined the IC_50_ of the binding inhibition of DHX36 to Z33 G4 to be 33 nM in the presence of **1** (Figure 3A and B). For concentrations higher than the IC_50_ we found binding inhibition increased by ∼20% in the range of 1–5 μM of **1**. In stark contrast, unwinding rates were reduced ∼70% in the same range (Figure 3C–E). The disparity between these values demonstrates that the decrease in unwinding activity is largely independent from binding inhibition of DHX36 by **1**. To further control for the possibility that ligands directly inhibit the enzyme, we measured the ATPase activity of DHX36 in the presence of poly(U), which was previously shown to stimulate DHX36 ATP consumption (37). ATP hydrolysis rates were not affected by any of the ligands making it unlikely that off-target ligand binding is responsible for DHX36 inhibition (Figure 4A and Supplementary Figure S12).

### DHX36 ATP-consumption is independent of substrate thermal stability

We next measured rates of ATP consumption by DHX36 in the presence of saturating concentrations of G4 substrates with different stabilities (Supplementary Figures S11 and S12). Previously, Hickson *et al*. demonstrated that the presence of G4-stabilizing ligands, such as various substituted acridines and porphyrins, reduced unwinding activity by the RecQ-like DNA G4-unwinding helicase, BLM (38). Additionally, other studies have shown that DNA intercalators reduce both the strand unwinding and ATPase activity of several helicases, including the *E. coli* UvrD/Rep helicase DNA helicase II, presumably by impeding the translocating helicase (39). We thus reasoned that ATP hydrolysis rates would similarly be proportionally reduced as a function of substrate stability, akin to the dependence of DHX36 unwinding rates on substrate stability. Contrary to our expectations, DHX36 ATP consumption was independent of the thermal stability of G4s. Neither the addition of G4-stabilizing ligands to Z33 (Figure 4B; *F*(4,25) = 0.12, ns) nor varying the number of G-tetrads within the G4 substrate (Figure 4C; *F*(3,20) = 0.05, ns) resulted in significantly different rates of ATP hydrolysis in the presence of DHX36. These observations allow us to propose a mechanism with which DHX36 unwinds G4 in a non-processive, highly localized strand separation fashion, which is consistent with its quantitative release from its G4 substrate upon addition of ATP (5).

## DISCUSSION

**Figure 5.**
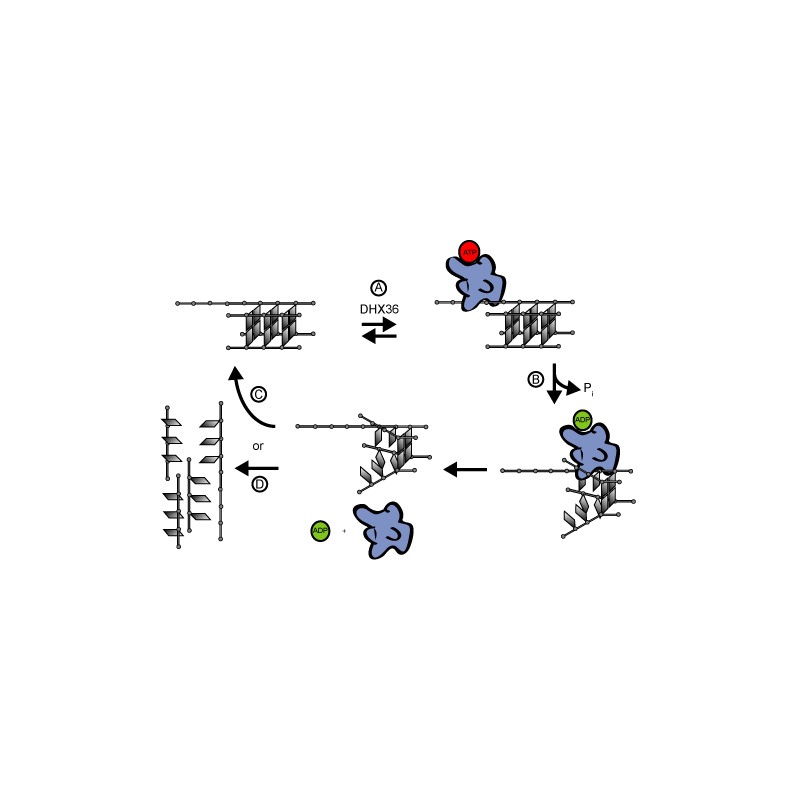
Model of DHX36-dependent unwinding of G4 substrates. (**A**) DHX36 binds to the 3′-tail of a G4 substrate in the presence or absence of ATP. (**B**) Upon ATP hydrolysis, DHX36 partially unwinds its G4 substrate and subsequently dissociates generating a destabilized G4. (**C**/**D**) Due to the non-processivity of DHX36, the G4 substrate is only partially unwound. The G4 either re-anneals or fully denatures depending on the stability of the destabilized G4 state. (Note: All DNA strands have 3′-overhangs, but for clarity the only strand depicted with a 3′-overhang is the one interacting with DHX36.)

Our results show that the DHX36 unwinding rate is sensitive to its G4 substrate thermal stability and that the ATP consumption of this helicase is independent of G4 stability. These observations suggest that DHX36 unwinds G4s via a non-processive, local strand-unwinding mechanism similar to that of the ds-specific DEAD-box helicase, eIF4A (15). DHX36 would first bind to the 3′-tail of its G4 substrate in an ATP-independent manner (Figure 5A). Upon ATP hydrolysis, the helicase destabilizes a number of G-tetrads and subsequently dissociates from its substrate generating a destabilized G4 (Figure 5B). Depending on its thermodynamic stability, the partially unwound G4 substrate can re-anneal (Figure 5C) or denature into its single-stranded components (Figure 5D). As the stability of the G4 substrate increases, the probability of non-productive strand unwinding increases resulting in an apparent decoupling between ATP hydrolysis and unwinding activity. It is also possible that the addition of cofactors may stimulate the processivity of DHX36 to translocate on its G4 substrate as observed for the closely related ds nucleic acids specific DEAH-box helicase DHX29 (40).

Our proposal that DHX36 uses an unwinding mechanism similar to that of ds-specific helicases is consistent with the pattern of structural conservation in the helicase core and the N/C terminal domains of DEAD-box and DEAH-box enzymes. The latter are highly variable and assist in diverse tasks such as specific nucleic acid binding, protein-protein interactions, as well as other functions (4). In DHX36 the element responsible for specific G4 recognition is located at its N-terminus (37). Conversely, the helicase core region of DHX36 contains two RecA-like domains that are highly conserved amongst members of the DExD/H-box helicase family (41). Thus, a conserved unwinding mechanism may reflect structural conservation. The ability for these helicases to use a conserved unwinding mechanism to process structurally distinct nucleic acids such as duplexes and G4s emphasizes the substrate versatility among DExD/H-box helicases. It is nevertheless surprising that substrates that are structurally very different, G4s and ds nucleic acids, are processed similarly by DEAD- and DEAH-box helicases.

Among translocating helicases, the dependence of helicase activity on substrate thermal stability is indicative of the unwinding mechanism (42,43). Unlike some DEAD and DEAH-box helicases, canonical helicases display an ability to translocate along a single-stranded nucleic acid substrate (19). By comparing the ratio of the double-stranded nucleic acid unwinding rate of a helicase on an AT-rich substrate (*V*_un_^AT^) to the unwinding rate on a GC-rich substrate (*V*_un_^GC^), translocating helicases can be separated into those that display an active or passive unwinding mechanism (42,43). Most DEAD-box helicases display an unwinding mechanism distinct from that of translocating helicases mentioned above (15,44), instead exhibiting highly local strand separation (19) and ATP hydrolysis rates independent of substrate stability or length (45). For example, the total ATP hydrolysis rate of CYT-19, a DEAD-box helicase, remains constant as its substrate (dsRNA) length increases (45). Our work shows that substrate stability is also useful in interrogating the mechanism of non-processive, local-acting helicases, despite their possessing an unwinding mechanism different from those of translocating helicases. Our conclusion is supported by Merrick *et al*. who attribute the observed ‘decoupling’ of eIF4A ATP consumption from dsRNA unwinding to the formation of non-productive complexes as a function of increasing substrate stability (15).

Whereas the aforementioned studies exclusively studied substrate stability by varying the sequence of the substrate nucleic acids, we also used G4-stabilizing ligands to study helicase mechanism. Even though the helicase core region exclusively contacts its substrate via the sugar-phosphate backbone (46), the accessory domains found on most DExD/H-box helicases may enable enzyme-specific substrate primary or secondary structure preferences (37). Using ligands to assess the impact of substrate stability removes the effect of primary sequence on helicase activity. However, a limitation of this technique is the ligand-mediated impact on helicase binding affinity, which we effectively control for in this study by comparing the degree of binding inhibition to unwinding inhibition (Figure 3).

The effect of G4-stabilizing ligands has been studied for several G4-unwinding helicases. The unwinding activity of WRN and BLM helicases were inhibited by several substituted acridine and porphyrin compounds (38). Contrary to our results, the rate of ATP consumption was affected by the addition of a G4-stabilizing ligand, which likely reflects the different mechanism of unwinding displayed by WRN and BLM compared to DHX36. The mechanistic differences are highlighted by Maizels *et al*., who propose that G4-stabilizing ligands result in a stalled BLM helicase that continues to consume ATP despite being stalled at a G4 structure (21). We attribute the difference in the proposed mechanism of action between BLM and DHX36 to the former belonging to the RecQ protein family, which consists of helicases with greater processivity. Importantly, Maizels *et al*. showed that stalled complexes exist even after the introduction of ATP, whereas no evidence of stalled complexes were found in this study. Thus, rather than a stalling mechanism, we propose that increasing substrate stability passively affects DHX36 activity by increasing the formation of non-productive complexes as outlined in Figure 5.

## CONCLUSION

In this study, we extend the growing knowledge of DEAH-box helicase unwinding mechanisms to DHX36, providing the first insights into the mechanism of a G4-unwinding DExD/H-box helicase. We demonstrate that the mechanism of G4 unwinding by DHX36 bears remarkable resemblance to that employed by homologous helicases whose function is to unwind dsRNA. This result suggests that G4-quadruplex helicases, such as DHX36, have evolved in order to selectively recognize G4-substrates without modifying their mechanism of unwinding. We show that G4-stabilizing ligands can be used to gain insight into helicase function by modulating substrate stability, an important parameter in mechanistic helicase studies (43). Previous studies have also documented the susceptibility of G4 helicases to G4-stabilizing ligands, such as WRN, BLM (20,21), Pif1 (47) and FANCJ (48). Our work furthermore emphasizes that G4 ligands retard helicase activity rather than quantitatively inhibit unwinding. Since G4 thermal stability affects the rate of helicase unwinding, we reason that G4 thermal stability likely affects the rate of certain biological processes such as replication and transcription. Rather than act as a binary on/off switch, our data suggest G4s may function as rheostats, a notion introduced in the investigation of riboswitches as genetic control elements (49). Given that G4s are linked with genomic instability due to the retardation of DNA Pol II (50) and result in decreased levels of transcription (51) as well as translation (52), G4 thermal stability may play a fine-tuning role in controlling gene expression.

## ACCESSION NUMBER

The coordinates have been deposited in the PDB under the accession code 4XK0.

## SUPPLEMENTARY DATA

Supplementary Data are available at NAR Online.

SUPPLEMENTARY DATA

## References

[1] Maizels N. (2006). Dynamic roles for G4 DNA in the biology of eukaryotic cells. Nat. Struct. Mol. Biol..

[2] Biffi G., Tannahill D., McCafferty J., Balasubramanian S. (2013). Quantitative visualization of DNA G-quadruplex structures in human cells. Nat. Chem..

[3] Biffi G., Di Antonio M., Tannahill D., Balasubramanian S. (2014). Visualization and selective chemical targeting of RNA G-quadruplex structures in the cytoplasm of human cells. Nat. Chem..

[4] Tanner N.K., Linder P. (2001). DExD/H Box RNA Helicases. Mol. Cell.

[5] Creacy S.D., Routh E.D., Iwamoto F., Nagamine Y., Akman S.A., Vaughn J.P. (2008). G4 resolvase 1 binds both DNA and RNA tetramolecular quadruplex with high affinity and is the major source of tetramolecular quadruplex G4-DNA and G4-RNA resolving activity in HeLa cell lysates. J. Biol. Chem..

[6] Giri B., Smaldino P.J., Thys R.G., Creacy S.D., Routh E.D., Hantgan R.R., Lattmann S., Nagamine Y., Akman S.A., Vaughn J.P. (2011). G4 resolvase 1 tightly binds and unwinds unimolecular G4-DNA. Nucleic Acids Res..

[7] Vaughn J.P., Creacy S.D., Routh E.D., Joyner-Butt C., Jenkins G.S., Pauli S., Nagamine Y., Akman S.A. (2005). The DEXH protein product of the DHX36 gene is the major source of tetramolecular quadruplex G4-DNA resolving activity in HeLa cell lysates. J. Biol. Chem..

[8] Huang W., Smaldino P.J., Zhang Q., Miller L.D., Cao P., Stadelman K., Wan M., Giri B., Lei M., Nagamine Y. (2012). Yin Yang 1 contains G-quadruplex structures in its promoter and 5′-UTR and its expression is modulated by G4 resolvase 1. Nucleic Acids Res..

[9] Booy E.P., Howard R., Marushchak O., Ariyo E.O., Meier M., Novakowski S.K., Deo S.R., Dzananovic E., Stetefeld J., McKenna S.A. (2013). The RNA helicase RHAU (DHX36) suppresses expression of the transcription factor PITX1. Nucleic Acids Res..

[10] Jain A., Bacolla A., Chakraborty P., Grosse F., Vasquez K.M. (2010). Human DHX9 helicase unwinds triple-helical DNA structures. Biochemistry.

[11] Murat P., Balasubramanian S. (2014). Existence and consequences of G-quadruplex structures in DNA. Curr. Opin. Genet. Dev..

[12] Chakraborty P., Grosse F. (2011). Human DHX9 helicase preferentially unwinds RNA-containing displacement loops (R-loops) and G-quadruplexes. DNA Repair.

[13] Linder P.P. (2006). Dead-box proteins: a family affair–active and passive players in RNP-remodeling. Nucleic Acids Res..

[14] Rogers G.W., Komar A.A., Merrick W.C. (2002). eIF4A: the godfather of the DEAD box helicases. Prog. Nucleic Acid Res. Mol. Biol..

[15] Rogers G.W., Richter N.J., Merrick W.C. (1999). Biochemical and kinetic characterization of the RNA helicase activity of eukaryotic initiation factor 4A. J. Biol. Chem..

[16] Rogers G.W., Lima W.F., Merrick W.C. (2001). Further characterization of the helicase activity of eIF4A. Substrate specificity. J. Biol. Chem..

[17] Yang Q., Jankowsky E. (2005). ATP- and ADP-dependent modulation of RNA unwinding and strand annealing activities by the DEAD-box protein DED1. Biochemistry.

[18] Liu F., Putnam A., Jankowsky E. (2008). ATP hydrolysis is required for DEAD-box protein recycling but not for duplex unwinding. Proc. Natl. Acad. Sci. U.S.A..

[19] Yang Q., Del Campo M., Lambowitz A.M., Jankowsky E. (2007). DEAD-box proteins unwind duplexes by local strand separation. Mol. Cell.

[20] Brosh R.M., Karow J.K., White E.J., Shaw N.D., Hickson I.D., Bohr V.A. (2000). Potent inhibition of werner and bloom helicases by DNA minor groove binding drugs. Nucleic Acids Res..

[21] Huber M.D., Lee D.C., Maizels N. (2002). G4 DNA unwinding by BLM and Sgs1p: substrate specificity and substrate-specific inhibition. Nucleic Acids Res..

[22] Karsisiotis A.I., Hessari N.M., Novellino E., Spada G.P., Randazzo A., Webba da Silva M. (2011). Topological characterization of nucleic acid G-quadruplexes by UV absorption and circular dichroism. Angew. Chem..

[23] Otwinowski Z., Minor W. (1997). Processing of X-ray diffraction data. Methods Enzymol..

[24] Schneider T.R., Sheldrick G.M. (2002). Substructure solution with SHELXD. Acta Crystallogr. D Biol. Crystallogr..

[25] Adams P.D., Afonine P.V., Bunkóczi G., Chen V.B., Davis I.W., Echols N., Headd J.J., Hung L.-W., Kapral G.J., Grosse-Kunstleve R.W. (2010). PHENIX: a comprehensive Python-based system for macromolecular structure solution. Acta Crystallogr. D Biol. Crystallogr..

[26] Emsley P., Cowtan K. (2004). Coot: model-building tools for molecular graphics. Acta Crystallogr. D Biol. Crystallogr..

[27] Akman S.A., Lingeman R.G., Doroshow J.H., Smith S.S. (1991). Quadruplex DNA formation in a region of the tRNA gene supF associated with hydrogen peroxide mediated mutations. Biochemistry.

[28] Mergny J.-L., De Cian A., Ghelab A., Saccà B., Lacroix L. (2005). Kinetics of tetramolecular quadruplexes. Nucleic Acids Res..

[29] Deng J., Xiong Y., Sundaralingam M. (2001). X-ray analysis of an RNA tetraplex (UGGGGU)4 with divalent Sr2+ ions at subatomic resolution (0.61 A). Proc. Natl. Acad. Sci. U.S.A..

[30] Sugimoto N., Ohmichi T., Sasaki M. (1996). The stability of DNA and RNA G-quartets. Nucleosides Nucleotides Nucleic Acids.

[31] Rodriguez R., Müller S., Yeoman J.A., Trentesaux C., Riou J.-F., Balasubramanian S. (2008). A novel small molecule that alters shelterin integrity and triggers a DNA-damage response at telomeres. J. Am. Chem. Soc..

[32] Rodriguez R., Miller K.M., Forment J.V., Bradshaw C.R., Nikan M., Britton S., Oelschlaegel T., Xhemalce B., Balasubramanian S., Jackson S.P. (2012). Small-molecule-induced DNA damage identifies alternative DNA structures in human genes. Nat. Chem. Biol..

[33] De Cian A., Cristofari G., Reichenbach P., de Lemos E., Monchaud D., Teulade-Fichou M.-P., Shin-ya K., Lacroix L., Lingner J., Mergny J.-L. (2007). Reevaluation of telomerase inhibition by quadruplex ligands and their mechanisms of action. Proc. Natl. Acad. Sci. U.S.A..

[34] De Cian A., DeLemos E., Mergny J.-L., Teulade-Fichou M.-P., Monchaud D. (2007). Highly efficient G-quadruplex recognition by bisquinolinium compounds. J. Am. Chem. Soc..

[35] Riou J.F., Guittat L., Mailliet P., Laoui A., Renou E., Petitgenet O., Mégnin-Chanet F., Hélène C., Mergny J.L. (2002). Cell senescence and telomere shortening induced by a new series of specific G-quadruplex DNA ligands. Proc. Natl. Acad. Sci. U.S.A..

[36] Bejugam M., Sewitz S., Shirude P.S., Rodriguez R., Shahid R., Balasubramanian S. (2007). Trisubstituted isoalloxazines as a new class of G-quadruplex binding ligands: small molecule regulation of c-kit oncogene expression. J. Am. Chem. Soc..

[37] Lattmann S., Giri B., Vaughn J.P., Akman S.A., Nagamine Y., Lattmann S., Giri B., Vaughn J.P., Akman S.A., Nagamine Y. (2010). Role of the amino terminal RHAU-specific motif in the recognition and resolution of guanine quadruplex-RNA by the DEAH-box RNA helicase RHAU. Nucleic Acids Res..

[38] Li J.L., Harrison R.J., Reszka A.P., Brosh R.M., Bohr V.A., Neidle S., Hickson I.D. (2001). Inhibition of the Bloom‘s and Werner's syndrome helicases by G-quadruplex interacting ligands. Biochemistry.

[39] George J.W., Ghate S., Matson S.W., Besterman J.M. (1992). Inhibition of DNA helicase II unwinding and ATPase activities by DNA-interacting ligands. Kinetics and specificity. J. Biol. Chem..

[40] Pisareva V.P., Pisarev A. V., Komar A.A., Hellen C.U.T., Pestova T.V. (2008). Translation initiation on mammalian mRNAs with structured 5′ UTRs requires DExH-box protein DHX29. Cell.

[41] Cordin O., Banroques J., Tanner N.K., Linder P. (2006). The DEAD-box protein family of RNA helicases. Gene.

[42] Manosas M., Xi X.G., Bensimon D., Croquette V. (2010). Active and passive mechanisms of helicases. Nucleic Acids Res..

[43] Byrd A.K., Matlock D.L., Bagchi D., Aarattuthodiyil S., Harrison D., Croquette V., Raney K.D. (2012). Dda helicase tightly couples translocation on single-stranded DNA to unwinding of duplex DNA: Dda is an optimally active helicase. J. Mol. Biol..

[44] Bizebard T., Ferlenghi I., Iost I., Dreyfus M. (2004). Studies on three E. coli DEAD-box helicases point to an unwinding mechanism different from that of model DNA helicases. Biochemistry.

[45] Chen Y., Potratz J.P., Tijerina P., Del Campo M., Lambowitz A.M., Russell R. (2008). DEAD-box proteins can completely separate an RNA duplex using a single ATP. Proc. Natl. Acad. Sci. U.S.A..

[46] Linder P., Jankowsky E. (2011). From unwinding to clamping — the DEAD box RNA helicase family. Nat. Rev. Mol. Cell Biol..

[47] Piazza A., Boulé J.-B., Lopes J., Mingo K., Largy E., Teulade-Fichou M.-P., Nicolas A. (2010). Genetic instability triggered by G-quadruplex interacting Phen-DC compounds in Saccharomyces cerevisiae. Nucleic Acids Res..

[48] Wu Y., Shin-ya K., Brosh R.M. (2008). FANCJ helicase defective in Fanconia anemia and breast cancer unwinds G-quadruplex DNA to defend genomic stability. Mol. Cell. Biol..

[49] Baird N., Kulshina N., D'Amaré A.F. (2010). Riboswitch function: Flipping the switch or tuning the dimmer. RNA Biol..

[50] Paeschke K., Bochman M.L., Garcia P.D., Cejka P., Friedman K.L., Kowalczykowski S.C., Zakian V.A. (2013). Pif1 family helicases suppress genome instability at G-quadruplex motifs. Nature.

[51] Siddiqui-Jain A., Grand C.L., Bearss D.J., Hurley L.H. (2002). Direct evidence for a G-quadruplex in a promoter region and its targeting with a small molecule to repress c-MYC transcription. Proc. Natl. Acad. Sci. U.S.A..

[52] Kumari S., Bugaut A., Huppert J.L., Balasubramanian S. (2007). An RNA G-quadruplex in the 5′ UTR of the NRAS proto-oncogene modulates translation. Nat. Chem. Biol..

