# Supplementary material for: Insights into the mechanism of a G-quadruplex-unwinding DEAH-box helicase
Source: Nucleic Acids Res. 2015 Feb 4;43(4):2223–31. doi: 10.1093/nar/gkv051 (PMC4344499; doi:10.1093/nar/gkv051)
Supplement: SUPPLEMENTARY DATA [file supp_43_4_2223__index.html]

Insights into the mechanism of a G-quadruplex-unwinding DEAH-box helicase — Insights into the mechanism of a G-quadruplex-unwinding DEAH-box helicase — SUPPLEMENTARY DATA 

# Insights into the mechanism of a G-quadruplex-unwinding DEAH-box helicase

## SUPPLEMENTARY DATA

**Files in this Data Supplement:**

- SUPPLEMENTARY DATA
