# Supplementary material for: Insights into the mechanism of a G-quadruplex-unwinding DEAH-box helicase
Source: Nucleic Acids Res. 2015 Feb 4;43(4):2223–31. doi: 10.1093/nar/gkv051 (PMC4344499; doi:10.1093/nar/gkv051)
Supplement: SUPPLEMENTARY DATA [file supp_gkv051_nar-03498-f-2014-File007.docx]

Insights into the mechanism of a G-quadruplex resolving DEAH-box helicase

Michael C. Chen, Pierre Murat, Keren Abecassis, Adrian R. Ferré-D’Amaré and Shankar Balasubramanian

**Supplementary Information**

**
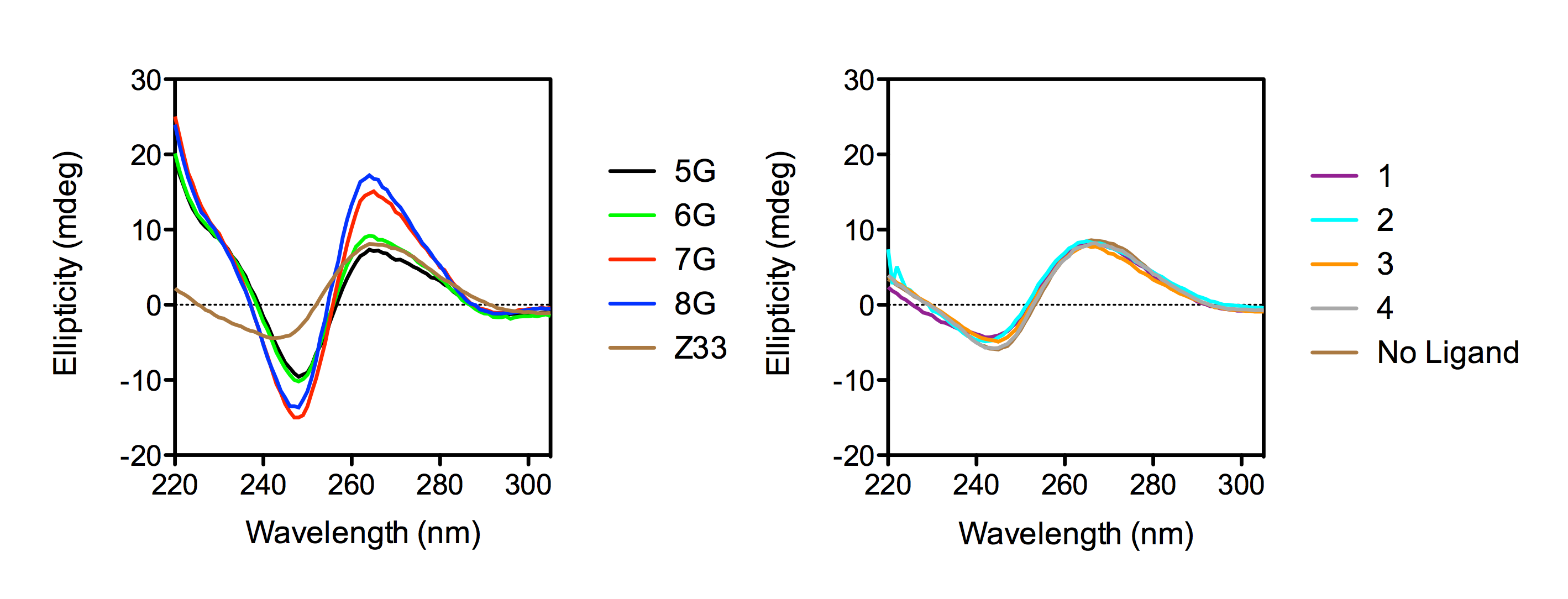
**

**Figure S1.** Circular dichroism spectra of the G4 substrates (spectra of 5G-8G and Z33 were acquired at a 20 and 10 μM strand concentration respectively and a concentration of 25 μM for compounds **1**-**4**) used in this study in K-Res buffer. All G4s correspond to parallel, intermolecular G4s as identified by peak maxima and minima.^3^


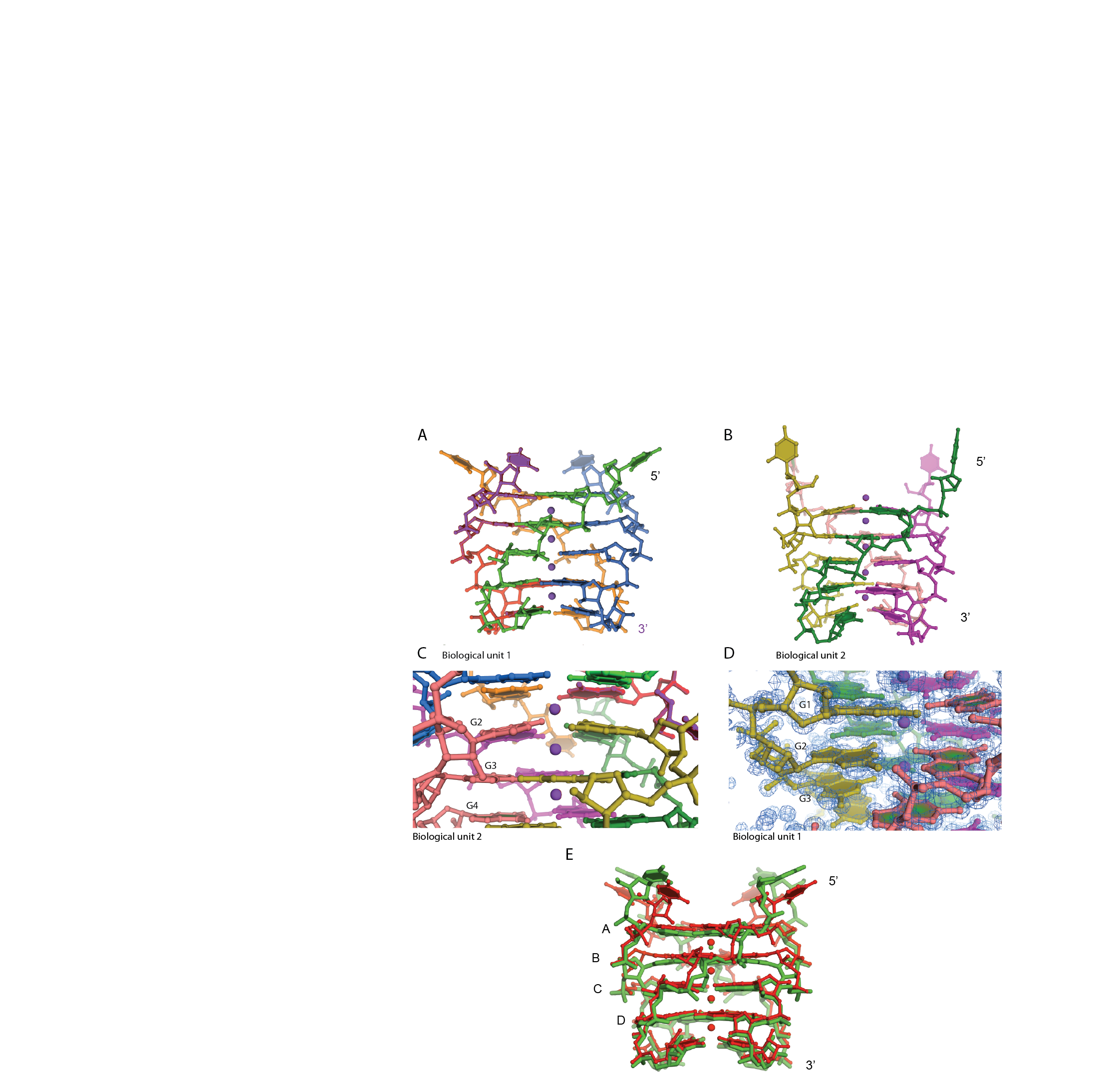


**Figure S2.** The structure of tetramolecular G4, r(UGGGGU)_4_, was determined to be a parallel, anti-glycosidic (for guanine residues) tetramolecular G4 in a potassium containing solution (PDBID 4XK0). (A) Model of biological unit 1, a parallel RNA tetramolecular G-quadruplex. (B) Model of biological unit 2, a parallel tetramolecular G-quadruplex. The 5'-end (top) of biological unit 2 stacks with the 5'-end (bottom) of biological unit 1 (A) to form a crystal contact. (C) Between the first and second tetrad of biological unit 2 (from 5'-end) the geometry of the guanine buckle angles are distinctly different likely due to crystal packing forces. (D) One tetrad of biological unit 1 with overlaid electron density shown at two standard deviations above the mean electron density of the unit cell of the final refined model. (E) Least squares superposition of biological unit 1 (red) with 1J8G^8^ (green), a 0.61 Å structure of tetramolecular G4, r(UGGGGU)_4_, crystallized in the presence of Sr^2+^. The similarity of the two structures indicates that the r(UGGGGU)_4_  G4 tetramolecular structure with Sr^2+^ (green spheres) is nearly identical to the structure with the physiological cation K^+^ (red spheres). However, the 1J8G quadruplex (green) contains Sr^2+^ in between G-quartet levels labeled A/B and C/D, whereas this structure (red) contains K^+^ at every G-quartet (A-D).

**
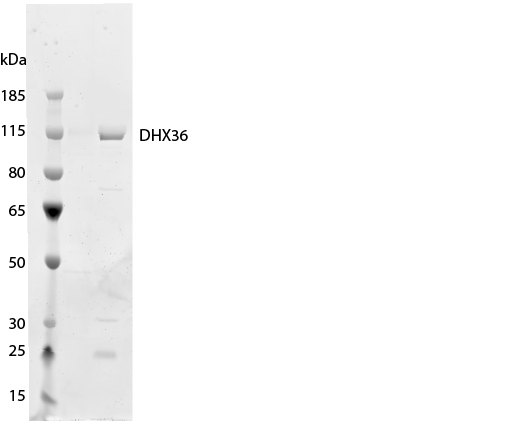
**

**Figure S3.** Purity of DHX36 (>90%) on SDS-PAGE after immobilized metal affinity chromatography. Band was further identified by trypsin-digest mass fingerprinting and western blot. Bands were visualized by Instant Blue coomassie stain (Expedeon, Inc.). Each band in the marker lane represents approximately 0.1 μg of protein.


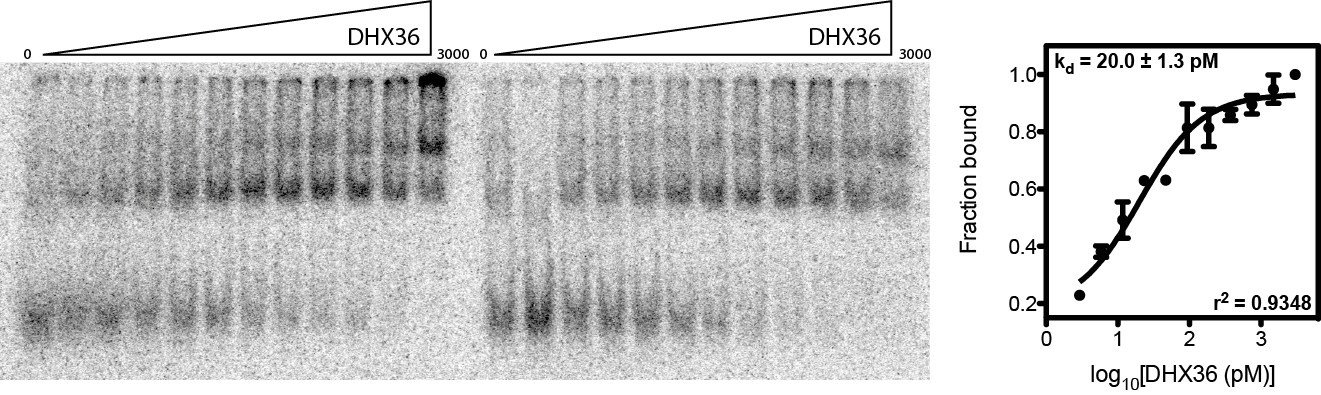


**Figure S4.** Binding of DHX36 to Z33 G4. DHX36 was incubated at indicated concentrations ([DHX36] = 0-3000 pM) with 10 pM Z33 G44 for 30 min at 37 °C in K-Res buffer without ATP. The apparent dissociation constant was determined to be 20.0 ± 1.3 pM as determined by a sigmoidal fit of the fraction Z33 G4 bound as a function of the log of DHX36 concentration. Measurements were performed in triplicate. Error represents the standard deviation of the measurements.


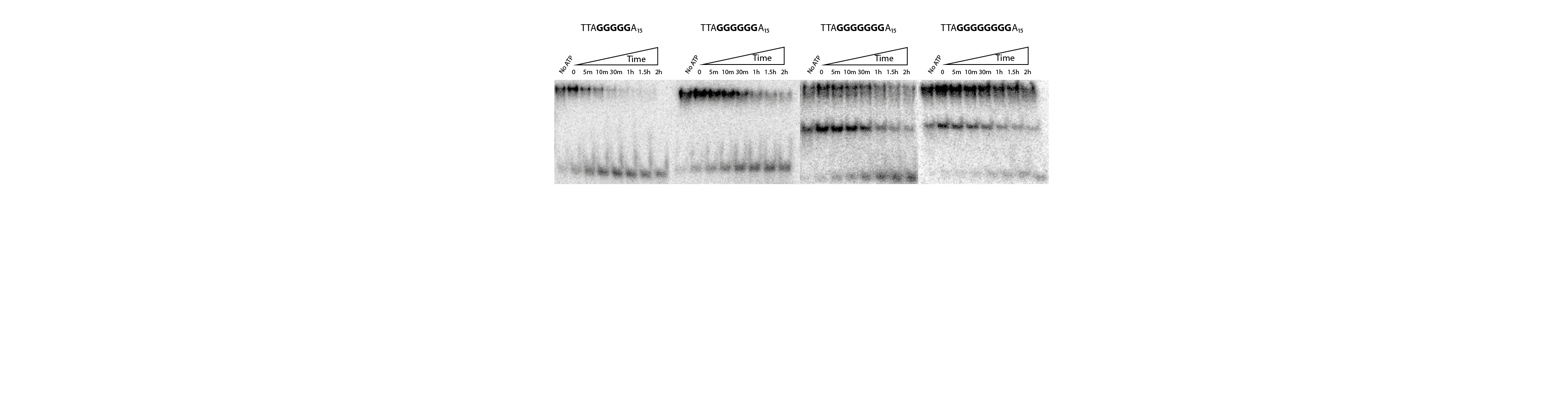


Unfolded G4

Folded G4

Folded G4

Unfolded G4

Higher order

**Figure S5.** Resolution of 5G-8G by DHX36 as a function of time. Quantifications are given in main text Figure 1. Experiments were performed in triplicate. Multiple bands present in gel for 7G and 8G may represent higher order nucleic acid structures. Higher order bands were not quantified.


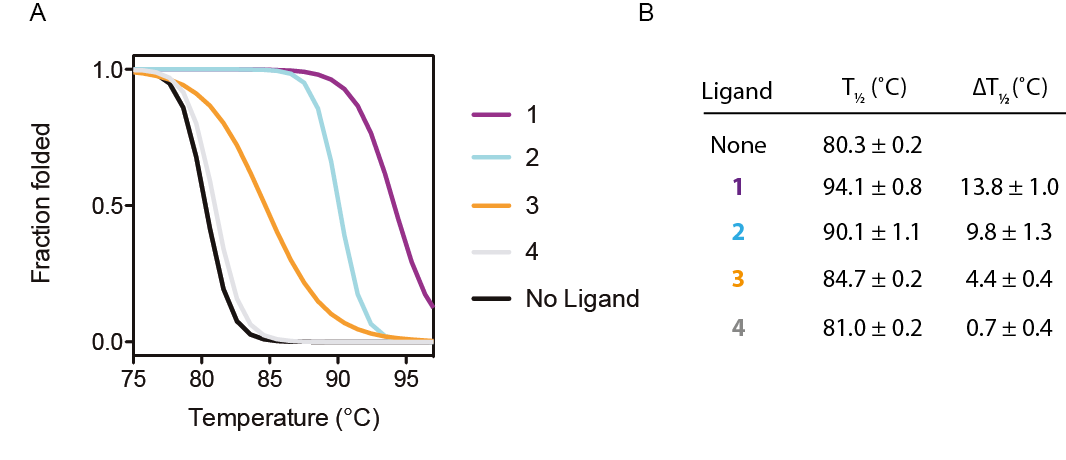


**Figure S6.** (A) Fraction folded curves calculated *via* CD spectroscopy melting experiment assuming a two-state transition. (B) Absolute and differential transition temperatures of Z33 G4 with and without ligands. The transition temperature was determined by first derivative analysis of calculated fraction folded curves shown in (A).


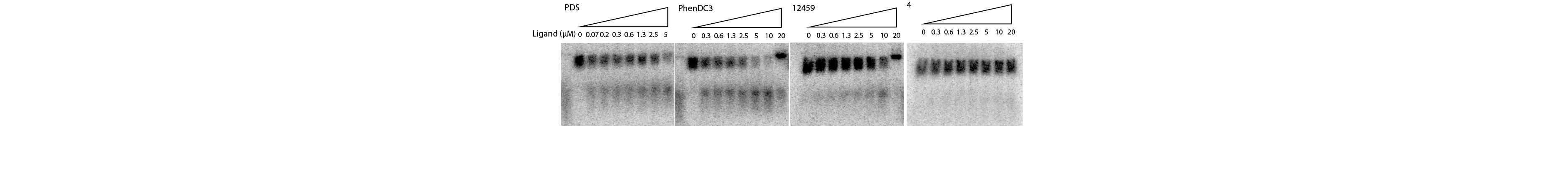


Figure S7. Binding inhibition of Z33-G4 with DHX36 in the presence of increasing ligand (1-4) concentration. Quantifications are given in Figure S9. Experiments were performed in triplicate.


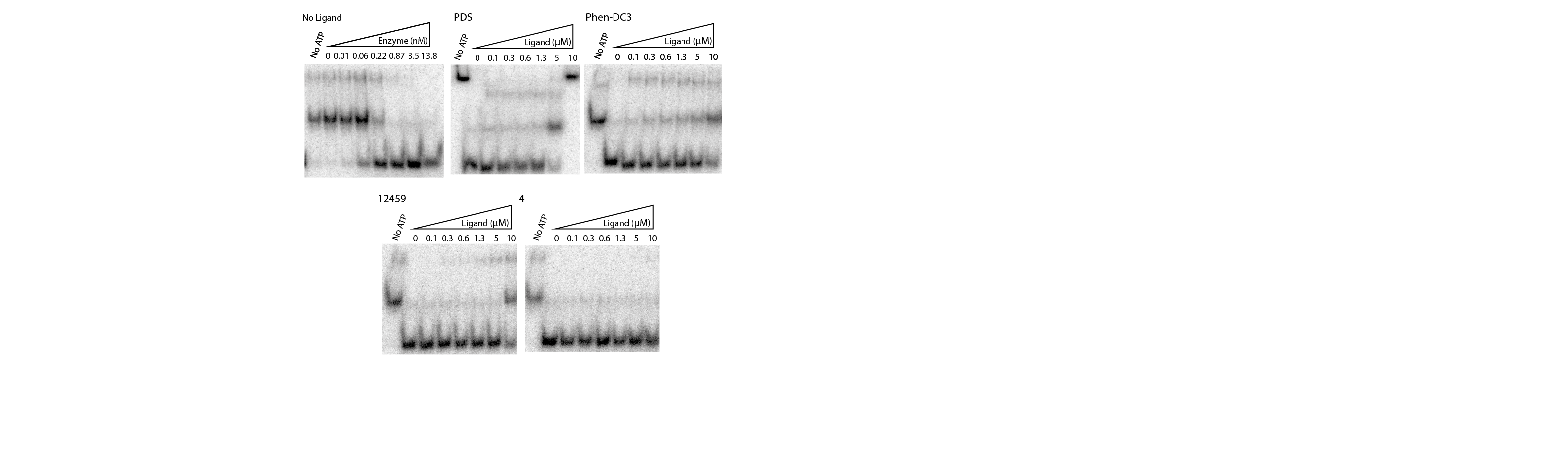


Higher order

Unfolded G4

Folded G4

Higher order

Folded G4

Unfolded G4

**Figure S8.** Resolution of Z33-G4 by DHX36 as a function of ligands **1**-**4** concentration. The resolution of Z33-G4 with different concentrations of DHX36 is given under the label “No Ligand.” The concentration of DHX36 in nanomolar is given above the gel. A concentration of 110 pM was used in subsequent resolution experiments with ligands. Quantifications are given in Figure S9. Experiments were performed in triplicate.


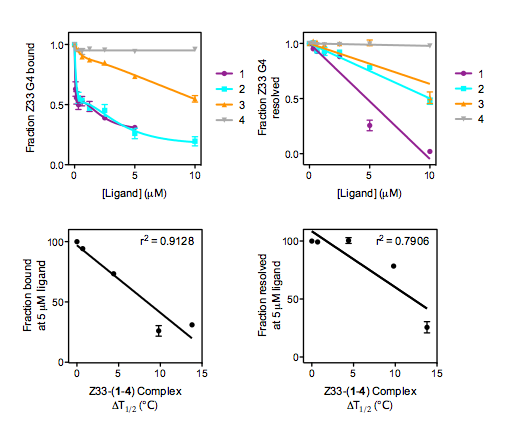


B

D

A

C

**Figure S9.** (A) A plot of the fraction of Z33-G4 bound by DHX36 when DHX36 (4 nM) was incubated with Z33 G4 in K-Res buffer as a function of ligand concentration. Ligands are identified by numbering scheme present in main text Figure 2A. (B) At a concentration of 5 μM ligand, the degree of binding inhibition as a function of Z33-ligand Δ*T*_1/2_ is presented. (C) A plot of the fraction of Z33-G4 bound by DHX36 when DHX36 (750 pM) was incubated with Z33 G4 in K-Res buffer supplemented with ATP as a function of ligand concentration. (D) At a concentration of 5 μM ligand, the degree of resolution inhibition as a function of Z33-ligand Δ*T*_1/2_ is presented. A poorer R-squared value is observed due to the deviation from linearity of total G4 resolution in the presence of compounds **3** and **4** in contrast to the linearity observed in binding inhibition (B).


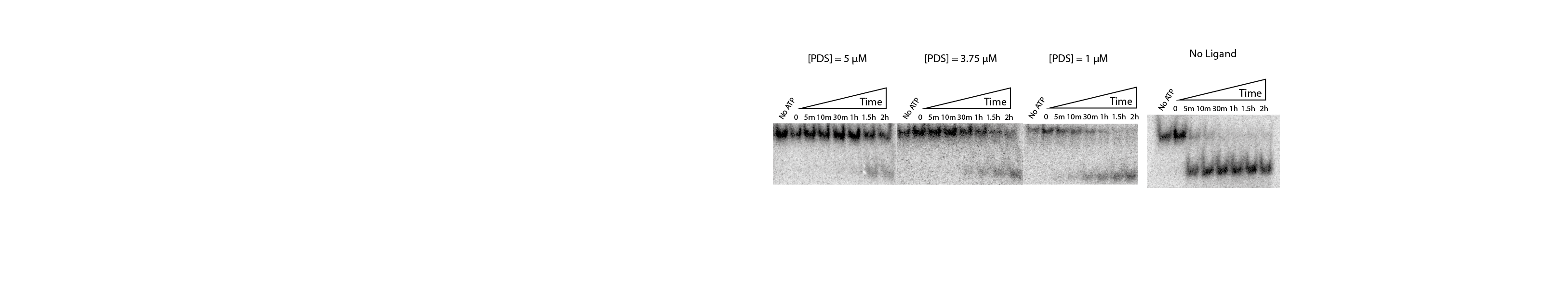


**Figure S10.** Resolution of Z33-G4 by DHX36 as a function of time and PDS concentration. Quantifications are given in Figure 3D and 3E. Experiments were performed in triplicate.


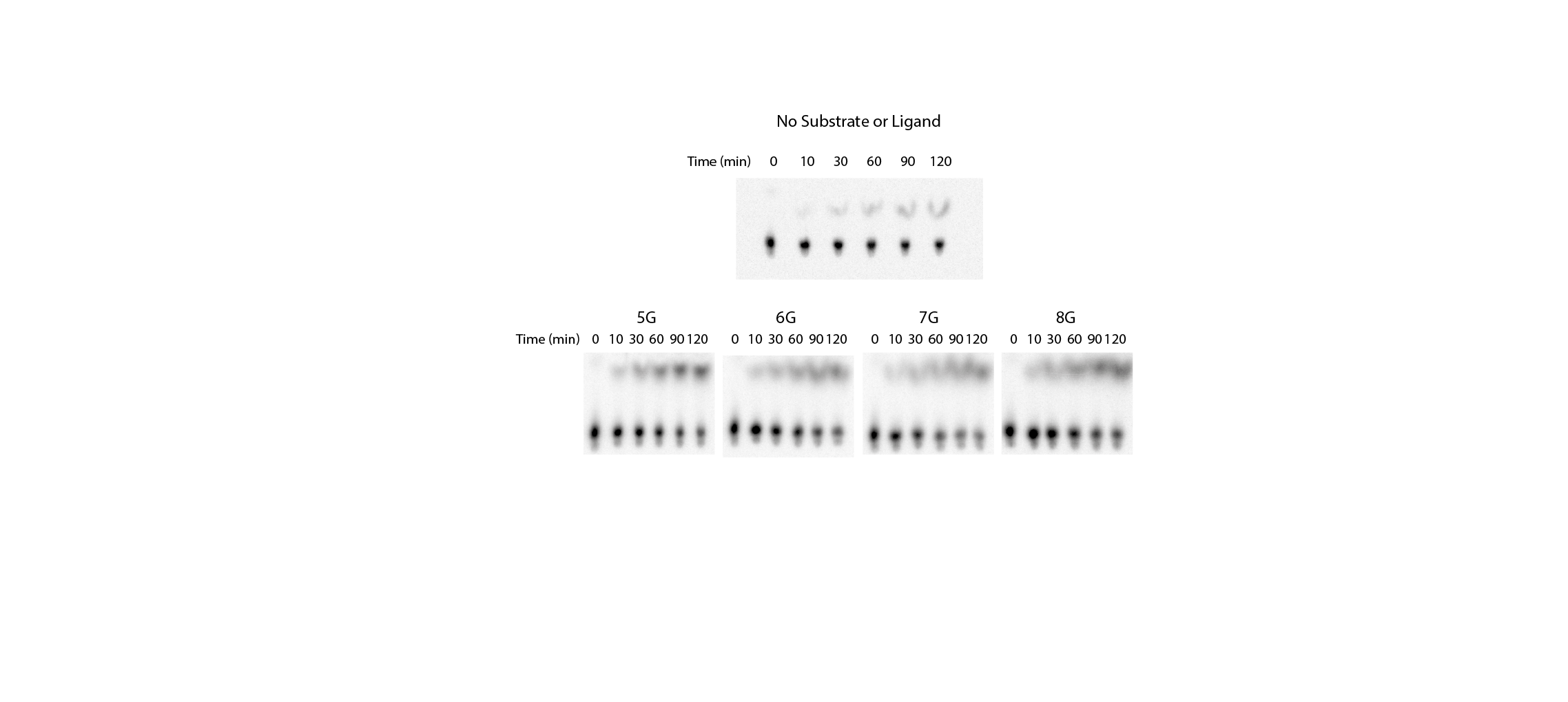


**Figure S11.** ATPase assay with DHX36 in the presence of no substrate/ligand or 5G-8G. Quantifications are given in the main text Figure 4. Experiments were performed in triplicate.


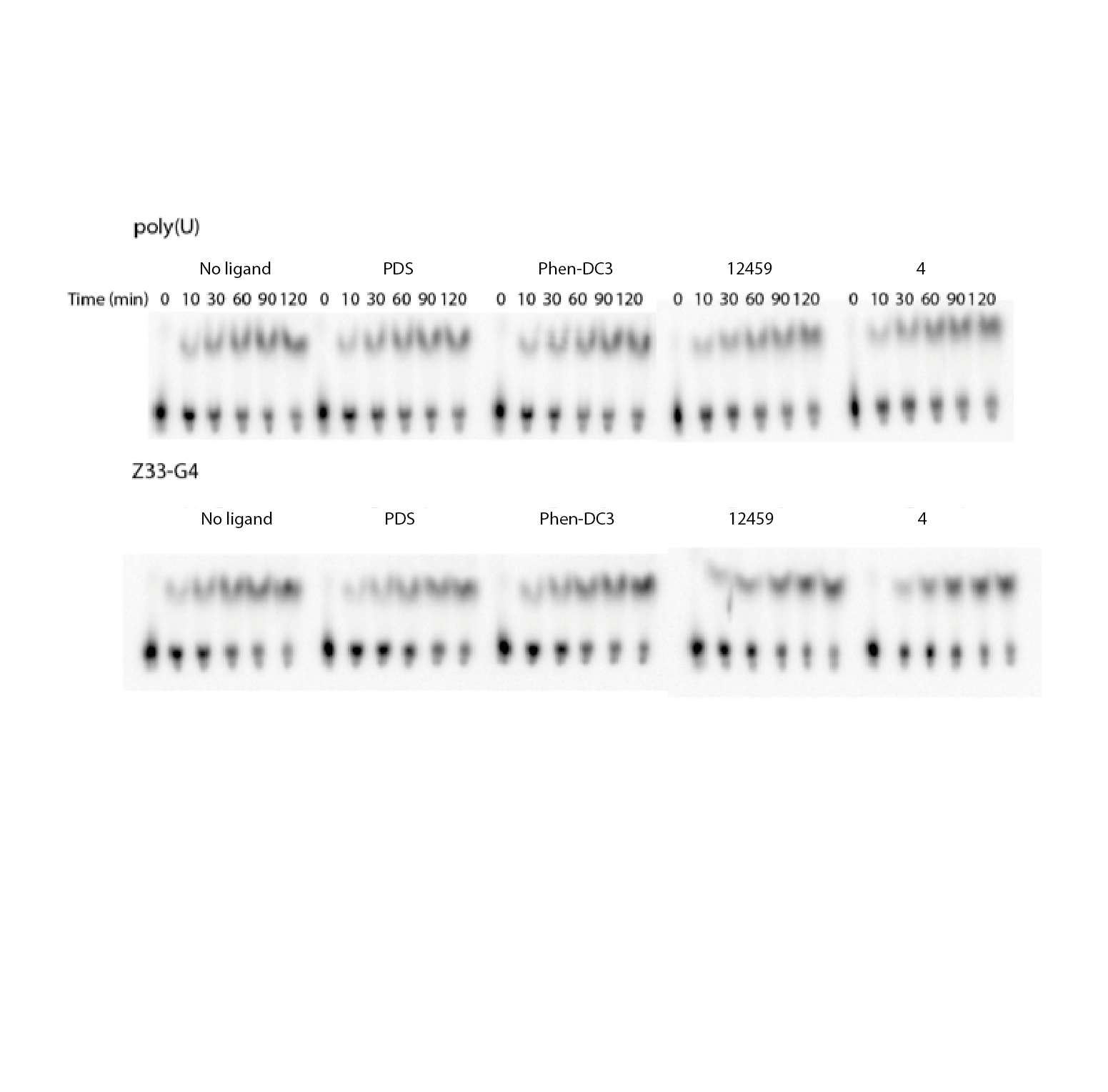


Figure S12. ATPase assay with DHX36 in the presence of poly(U) or Z33 G4. Quantifications are given in the main text Figure 4. Experiments were performed in triplicate.

**Table S1.** Data collection, phasing, and refinement statistics

| **Data collection** |  |
| --- | --- |
| Wavelength (Å) | 0.8266 |
| Space Group | *P*42_1_2 |
| Cell dimensions (Å) | *a* = 33.119, *b* = 33.119, *c* = 54.827 |
| Resolution (Å) | 50.0 - 1.09 (1.09 - 1.07)^*^ |
| R_merge_ | 0.074 (0.137)^*^ |
| <*I*>/<σ(*I*)> | 19.06 (15.76)^*^ |
| Completeness (%) | 99.6 (95.9)^*^ |
| Redundancy | 7.1 (5.7)^*^ |
| **Phasing** |  |
| Number of atoms in substructure^†^ | 14 |
| <*f.o.m*> 50.0 – 1.1 Å, before d.m. | 0.687 |
| **Refinement** |  |
| Resolution (Å) | 21.54 - 1.08 (1.09 -1.08)^*^ |
| Unique reflections | 25285 (931)^*^ |
| *R*_work_/*R*_free_ | 14.4 (20.6)^*^ / 14.4 (20.6)^*^ |
| No. atoms |  |
| RNA | 387 |
| Ligand/ion | 12 |
| Water | 62 |
| Mean *B*-factors (Å^2^) |  |
| RNA | 6.75 |
| Ligand/ion | 5.33 |
| Water | 19.31 |
| r.m.s. deviations |  |
| Bond lengths (Å) | 0.004 |
| Bond angles (°) | 0.894 |

^*^Highest resolution shell (1.09-1.08 Å). ^†^See materials and methods.
